# A Novel Technique for Achieving the Approximated ISI at the Receiver for a 16QAM Signal Sent via a FIR Channel Based Only on the Received Information and Statistical Techniques

**DOI:** 10.3390/e22060708

**Published:** 2020-06-26

**Authors:** Hadar Goldberg, Monika Pinchas

**Affiliations:** Department of Electrical and Electronic Engineering, Ariel University, Ariel 40700, Israel; hadare123@gmail.com

**Keywords:** maximum entropy density approximation, Edgeworth expansion, lagrange multipliers, generalized Gaussian distribution (GGD), inter-symbol-interference (ISI)

## Abstract

A single-input-multiple-output (SIMO) channel is obtained from the use of an array of antennas in the receiver where the same information is transmitted through different sub-channels, and all received sequences are distinctly distorted versions of the same message. The inter-symbol-interference (ISI) level from each sub-channel is presently unknown to the receiver. Thus, even when one or more sub-channels cause heavy ISI, all the information from all the sub-channels was still considered in the receiver. Obviously, if we know the approximated ISI of each sub-channel, we will use in the receiver only those sub-channels with the lowest ISI level to get improved system performance. In this paper, we present a systematic way for obtaining the approximated ISI from each sub-channel modelled as a finite-impulse-response (FIR) channel with real-valued coefficients for a 16QAM (16 quadrature amplitude modulation) source signal transmission. The approximated ISI is based on the maximum entropy density approximation technique, on the Edgeworth expansion up to order six, on the Laplace integral method and on the generalized Gaussian distribution (GGD). Although the approximated ISI was derived for the noiseless case, it was successfully tested for signal to noise ratio (SNR) down to 20 dB.

## 1. Introduction

Let us consider for a moment the digital communication case where during transmission, a source signal undergoes a convoluted distortion between its symbols and the channel impulse response. This distortion is referred to as the inter-symbol-interference (ISI) which causes harmful distortions, and presents a major difficulty in the recovery process [1]. In order to recover the sent sequence, a single-input-multiple-output (SIMO) blind adaptive equalization method may be applied where several receive antennas are used at the receiver side [2,3,4,5]. All the received sequences from the different receive antennas will be distinctly distorted versions of the same message (SIMO configuration) [6]. The information from all the received antennas is driven to an array of blind adaptive equalizers (SIMO case [7,8]) that outputs the estimated sent sequence. Until now, the information from all the received antennas was used in the recovery process even if one or more received piece of information was heavily damaged due to the channel because there was no way to know if the received information from a specific received antenna contained heavy ISI. Obviously, if we can estimate the initial ISI from the different receive antennas, we will take to the recovery process only the information from those receive antennas having the lowest initial ISI. Thus, we may acquire a faster convergence speed of the blind adaptive equalizer, which may lead to a faster recovery process of the sent sequence on one hand, and on the other hand may leave the system with improved performance from the residual ISI point of view, which may lead to a lower bit-error-rate (BER) of the recovered sent sequence.

In this paper, we propose for the 16 quadrature amplitude modulation (16QAM) input constellation case, a systematic way to obtain the approximated initial ISI obtained from each receive antenna where each path from the sent antenna to the specific receive antenna is modeled as a finite-impulse-response (FIR) channel with real-valued coefficients. For the noiseless case, the equalized output signal is built up from the recovered sent signal in addition to an error called the convolutional noise [9]. This convolutional noise is very high at the early stages of the iterative deconvolution process (equalization process) and is considered as small at the latter stages of the iterative deconvolution process where the process is close to optimality [10]. A high convolutional noise means that the equalizer leaves the system with a high residual ISI, whereas a small convolutional noise at the equalized output means that the equalizer leaves the system with a relative small residual ISI. At the early stages of the deconvolution process, the convolutional noise probability density function (pdf) is more a uniform distribution [10,11], whereas at the latter stages of the iterative deconvolution process, the convolutional noise probability density function (pdf) is approximately Gaussian [10,11]. Thus, during the iterative deconvolution process, the shape of the convolutional noise pdf changes.

The main idea in our systematic approach for obtaining the approximated initial ISI is using two different approaches for estimating the real part of the equalized output signal pdf for the noiseless case, and then comparing between them. On the one hand, the real part of the equalized output signal pdf is approximated with the maximum entropy density approximation technique [12,13,14,15] with Lagrange multipliers up to order four. On the other hand, the real part of the equalized output signal pdf is calculated using Bayes rules where the conditional pdf of the real part of the equalized output signal, given the real part of the input signal is obtained via the real part of the convolutional noise pdf, and the real part of the input sequence pdf that is approximated with the maximum entropy density approximation technique [12,13,14,15] with Lagrange multipliers up to order four. Please note that the Lagrange multipliers of the real part of the source and equalized output signal pdf are not the same. The real part of the convolutional noise pdf is approximated with the generalized Gaussian distribution (GGD) [16], where changes in the shape parameter of the GGD presentation change the shape of the pdf, which may have a Laplacian or double exponential distribution, a Gaussian distribution or a uniform distribution for a shape parameter equal to one, two and infinity respectively [16]. Since the integral of the real part of the input signal pdf multiplied by the conditional pdf of the real part of the equalized output signal given the real part of the input signal is a difficult task to carry out due to the fact that the shape parameter which appears at the exponent may be a fraction, the GGD is approximated with the Edgeworth expansion [17,18,19] up to order six where the different moments needed for the Edgeworth expansion are calculated according to [16]. Finally, this integral (the integral of the approximated real part of the input signal pdf multiplied by the approximated conditional pdf of the real part of the equalized output signal, given the real part of the input signal) is carried out with the Laplace Integral method [12,20].

Although the approximated ISI was derived for the noiseless case, it was successfully tested for signal to noise ratio (SNR) down to 20 dB.

The paper is organized as follows: After having described the systematic way for obtaining the approximated initial ISI in Section 2, we introduce in Section 3 our simulation results. Finally, the conclusion is presented in Section 4.

## 2. The Systematic Approach for Getting the Approximated Initial ISI

**Figure 1:**
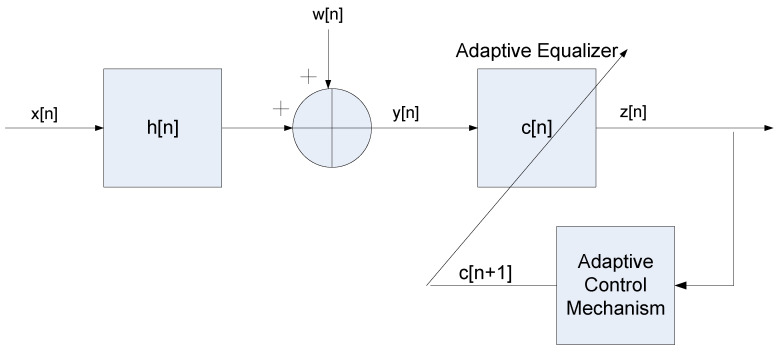
Block diagram of the system.

In this section, we present our systematic approach for achieving the approximated initial ISI for the 16QAM constellation input for the noiseless case. Let us consider the following system (Figure 1), where we make the following assumptions:

1. The input sequence x[n] is a **16QAM source** (a modulation using ± {1,3} levels for in-phase and quadrature components) which can be written as $x\left[n\right]=x_{r}\left[n\right]+jx_{i}\left[n\right]$ where $x_{r}\left[n\right]$ and $x_{i}\left[n\right]$ are the real and imaginary parts of x[n] respectively. $x_{r}\left[n\right]$ and $x_{i}\left[n\right]$ are independent and E[x[n]]=0.
2. The unknown channel h[n] is a possibly nonminimum phase linear time-invariant filter in which the transfer function has no “deep zeros”; namely, the zeros lie sufficiently far from the unit circle.
3. The filter c[n] is a tap-delay line.
4. The channel noise w[n] is an additive Gaussian white noise.

The equalizer’s input sequence y[n] is given by:

(1) y[n]=x[n]∗h[n]+w[n]

where “∗” stands for the convolutional operation. With the help of (1), the equalized output sequence can be written as:

(2) $$z\left[n\right]=y\left[n\right]*c\left[n\right]=\left(x[n]*h[n]+w[n]\right)*c\left[n\right]=x\left[n\right]*\tilde{s}\left[n\right]+\tilde{w}\left[n\right]$$

with

(3) $$\tilde{s}\left[n\right]=c\left[n\right]*h\left[n\right]=\delta \left[n\right]+\xi \left[n\right]$$

where ξ[n] stands for the difference (error) between the ideal and the used value for c[n] following (4), δ is the Kronecker delta function and $\tilde{w}\left[n\right]=w\left[n\right]*c\left[n\right]$. The equalizer’s coefficients are updated according to [21]:

(4) $$\underline{c}\left[n+1\right]=\underline{c}\left[n\right]-\mu \frac{\partial F[n]}{\partial z[n]}{\underline{y}*}^{}\left[n\right]$$

where ${\left(\right)}^{*}$ is the conjugate operation, μ is the step-size parameter, F[n] is the cost function and $\underline{c}\left[n\right]$ is the equalizer vector where the input vector is $\underline{y}\left[n\right]={\left[y\left[n\right]\ldots y\left[n-N+1\right]\right]}^{T}$. The operator ${\left(\right)}^{T}$ denotes for transpose of the function () and *N* is the equalizer’s tap length. In this paper we use Godard’s algorithm [22]. Thus we have:

(5) $$\frac{\partial F[n]}{\partial z[n]}=\left({\left|z[n]\right|}^{2}-\frac{E\left[{\left|x[n]\right|}^{4}\right]}{E\left[{\left|x[n]\right|}^{2}\right]}\right)z\left[n\right]$$

where |()| stands for the absolute value of (). The ISI is often used as a measure of performance in equalizers’ applications, defined by:

(6) $$ISI=\frac{\sum_{\tilde{m}}|\tilde{s}\left[\tilde{m}\right]{|}^{2}-{\left|\tilde{s}\right|}_{max}^{2}}{|\tilde{s}{|}_{max}^{2}}$$

where $|\tilde{s}{|}_{max}$ is the component of $\tilde{s}$, given in (3), having the maximal absolute value. Figure 2 shows the equalizer’s performance from the residual ISI point of view as a function of the iteration number for the noiseless and 16QAM input constellation case. Please note that at each iteration number, corresponding to a specific ISI level, the convolutional noise can be obtained with the help of (2) and (3) via

(7) p[n]=z[n]−x[n]

where p[n]=x[n]∗ξ[n]. At the latter stages of the iterative deconvolution process when the residual ISI is relative low, the input sequence x[n] and the convolutional noise p[n] may be considered as independent [10]. Thus we may write for the noiseless case:

(8) $$\sigma_{p[n]}^{2}=\sigma_{z[n]}^{2}-\sigma_{x[n]}^{2}$$

which can be written with the help of (2) for the noiseless case as:

(9) $$\sigma_{p[n]}^{2}=\sigma_{x[n]}^{2}\sum_{\tilde{m}}{\left|{\tilde{s}}_{\tilde{m}}\left[n\right]\right|}^{2}-\sigma_{x[n]}^{2}=\sigma_{x[n]}^{2}\left(\sum_{\tilde{m}}{\left|{\tilde{s}}_{\tilde{m}}\left[n\right]\right|}^{2}-1\right)$$

Based on (6) and (9) we may write for the noiseless case:

(10) $$\sigma_{p[n]}^{2}=\sigma_{x[n]}^{2}ISI\;\;\mathrm{for}\;|\tilde{s}{|}_{max}=1$$

Please note that at the latter stages of the iterative deconvolution process when the residual ISI is relative low, Godard’s [22] algorithm leaves the system with $|\tilde{s}{|}_{max}$ approximately equal to one.

**Theorem** **1.** *For the noiseless case, the approximated ISI can be calculated via the following equations:*

(11) $$\begin{array}{c} a+Q\left(ISI\right)\left(8a\lambda_{4}^{2}m_{6}+8a\lambda_{2}\lambda_{4}m_{4}+\left(2a\lambda_{2}^{2}+6a\lambda_{4}\right)m_{2}+b+a\lambda_{2}\right)+\\ Q^{2}{\left(ISI\right)}^{2}\left(32a\lambda_{4}^{4}m_{12}+64a\lambda_{2}\lambda_{4}^{3}m_{10}+\left(48a\lambda_{2}^{2}\lambda_{4}^{2}+144a\lambda_{4}^{3}\right)m_{8}+\right.\\ \left.\left(16a\lambda_{2}^{3}\lambda_{4}+168a\lambda_{2}\lambda_{4}^{2}+24b\lambda_{4}^{2}\right)m_{6}+\left(102a\lambda_{4}^{2}+60a\lambda_{2}^{2}\lambda_{4}+\right.\right.\\ \left.\left.2a\lambda_{2}^{4}+24b\lambda_{2}\lambda_{4}\right)m_{4}+\left(18b\lambda_{4}+6a\lambda_{2}^{3}+42a\lambda_{2}\lambda_{4}+6b\lambda_{2}^{2}\right)m_{2}+\right.\\ \left.1.5a\lambda_{2}^{2}+3b\lambda_{2}+3a\lambda_{4}+3c\right)=\\ 1+\left(\Delta \lambda_{2}m_{2}+\Delta \lambda_{4}m_{4}\right)+\frac{1}{2}\left(\Delta \lambda_{2}^{2}m_{4}+2\Delta \lambda_{2}\Delta \lambda_{4}m_{6}+\Delta \lambda_{4}^{2}m_{8}\right)+\\ \frac{1}{6}\left(\Delta \lambda_{2}^{3}m_{6}+3\Delta \lambda_{2}^{2}\Delta \lambda_{4}m_{8}+3\Delta \lambda_{2}\Delta \lambda_{4}^{2}m_{10}+\Delta \lambda_{4}^{3}m_{12}\right) \end{array}$$

where

(12) $$\begin{array}{c} a=\left(\frac{7w}{16}-\frac{r}{48}\right)\;\;\;\;b=\left(\frac{1}{\sigma_{p_{r}}^{2}}\right)\left(-\frac{19w}{16}+\frac{r}{16}+\frac{21}{8}\right)\\ c=\left(\frac{1}{\sigma_{p_{r}}^{4}}\right)\left(\frac{17w}{48}-\frac{r}{48}-\frac{3}{4}\right) \end{array}$$

(13) $$\sigma_{p_{r}}^{2}=Q\left(ISI\right)\;\;Q\;\mathrm{is}\;a\;\mathrm{predefined}\;\mathrm{parameter}$$

(14) $$\begin{array}{c} r=\frac{1}{\Gamma {\left(\frac{3}{\rho }\right)}^{3}}\Gamma {\left(\frac{1}{\rho }\right)}^{2}\Gamma \left(\frac{7}{\rho }\right)\;\;\;\;w=\frac{1}{\Gamma {\left(\frac{3}{\rho }\right)}^{2}}\Gamma \left(\frac{1}{\rho }\right)\Gamma \left(\frac{5}{\rho }\right)\;\;\;\;m_{k}=E\left[z_{r}^{k}\right] \end{array}$$

(15) $$\begin{array}{c} \rho ≅-1.1938\;\times 10^{-5}{\left(ISI_{dB}\right)}^{4}-7.3370\;\times 10^{-4}{\left(ISI_{dB}\right)}^{3}-0.0146{\left(ISI_{dB}\right)}^{2}-0.0693\left(ISI_{dB}\right)+2.6266\\ ISI_{dB}=10log_{10}\left(ISI\right) \end{array}$$

(16) $$\Delta \lambda_{2}={\tilde{\lambda }}_{2}-\lambda_{2};\;\;\;\;\Delta \lambda_{4}={\tilde{\lambda }}_{4}-\lambda_{4}$$

and where ${\tilde{\lambda }}_{2}$, $\lambda_{2}$, ${\tilde{\lambda }}_{4}$ and $\lambda_{4}$ were derived via [12]:

(17) $$\begin{array}{c} 1+4\lambda_{2_{i}}m_{2_{i}}+8\lambda_{4_{i}}m_{4_{i}}=0\\ 3m_{2_{i}}+8\lambda_{4_{i}}m_{6_{i}}+4\lambda_{2_{i}}m_{4_{i}}=0 \end{array}$$

where $m_{k_{i}}$, $\lambda_{2_{i}}$ and $\lambda_{4_{i}}$ for (i=1,2) were defined in this paper as:

(18) $$\begin{array}{c} m_{k_{1}}=E\left[z_{r}^{k}\right]=m_{k};\;\;\;\lambda_{2_{1}}={\tilde{\lambda }}_{2};\;\;\;\lambda_{4_{1}}={\tilde{\lambda }}_{4}\\ m_{k_{2}}=E\left[x_{r}^{k}\right];\;\;\;\;\;\lambda_{2_{2}}=\lambda_{2};\;\;\;\;\lambda_{4_{2}}=\lambda_{4} \end{array}$$

and where Γ and E[·] stand for the Gamma function and expectation operator respectively.

**Proof of** **Theorem 1.** At first, we will show the steps that led us to (15). After that, we will show and explain how we derived the rest of the above equations.

For the 16QAM constellation, the real and imaginary parts of x[n] are independent. Thus, in the following we will focus only on the real part of the input and equalized output signal. In addition, for simplicity, we use $x_{r}$, $z_{r}$ and $p_{r}$ for the real parts of x[n], z[n] and p[n], respectively. Therefore, based on (7) we have for the noiseless case:

(19) $$p_{r}=z_{r}-x_{r}$$

Based on (10) and (19) we may write for the latter stages of the iterative deconvolution process when the residual ISI is relative low and for the noise less case that:

(20) $$\begin{array}{c} \sigma_{p[n]}^{2}=2\sigma_{p_{r}}^{2}=\sigma_{x[n]}^{2}ISI=2\sigma_{x_{r}}^{2}ISI\;\;\mathrm{for}|\tilde{s}{|}_{max}=1\\ ⇓\\ \sigma_{p_{r}}^{2}=\sigma_{x_{r}}^{2}ISI\;\;\mathrm{for}|\tilde{s}{|}_{max}=1 \end{array}$$

**Figure 3:**
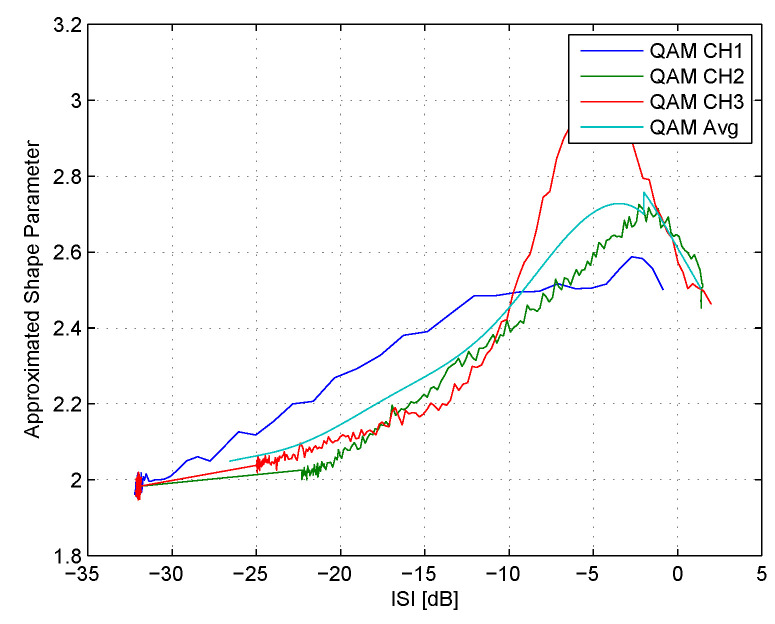
The approximated shape parameter as a function of the residual ISI in dB units for the 16QAM constellation input sent via three different channels (CH1, CH2 and CH3) for the noiseless case. The parameters *L* and *t* were set to 300 and 1000 respectively. The simulation length (the total number of iteration number) used for CH1, CH2 and CH3 was set to 500414, 190414 and 190414 respectively.

Based on (20), we have for the noiseless case that at the latter stages of the iterative deconvolution process when the residual ISI is relative low, $Q=\sigma_{x_{r}}^{2}$. However, (20) does not hold at the early stages of the iterative deconvolution process, wherein the input sequence x[n] is dependent with the convolutional noise p[n]. Thus, at the early stages of the iterative deconvolution process, *Q* may be different from $\sigma_{x_{r}}^{2}$. Now, if we carry out *L* Monte Carlo trials of the equalizer’s performance from the residual ISI point of view, then we have at each iteration number, corresponding to a specific ISI level, *L* samples of the convolutional noise. In the following, we denote $p_{r_{i,ISI_{j}}}$ as the real part of the convolutional noise corresponding to iteration number *j* with residual ISI of $ISI_{j}$, belonging to the *i*-th Monte Carlo trial. According to [16], the generalized Gaussian function ratio (ggfr) is given by:

(21) $$M\left(\rho \right)=\frac{{\left(E\left[|p_{r}|\right]\right)}^{2}}{E[p_{r}^{2}]}=\frac{\Gamma^{2}\left(\frac{2}{\rho }\right)}{\Gamma \left(\frac{1}{\rho }\right)\Gamma \left(\frac{3}{\rho }\right)}$$

where ρ is the shape parameter. The relationship between the approximated shape parameter (defined in the following as $\tilde{\rho }$) and approximated $M\left(\rho \right)$ (defined in the following as $\tilde{M}\left(\rho \right)$) is given by [16]:

(22) $$\tilde{\rho }=\left\{\begin{array}{cc} 2\frac{\ln\frac{27}{16}}{\ln\frac{3}{4{\tilde{M}}^{2}\left(\rho \right)}} & \mathrm{if}\tilde{M}\left(\rho \right)\varepsilon \left(0,0.131246\right)\\ \frac{1}{2a_{1}}\left(-a_{2}+\sqrt{a_{2}^{2}-4a_{1}a_{3}+4a_{1}\tilde{M}\left(\rho \right)}\right) & \mathrm{if}\tilde{M}\left(\rho \right)\varepsilon \left[0.131246,0.448994\right)\\ \frac{1}{2b_{3}\tilde{M}\left(\rho \right)}\left(b_{1}-b_{2}\tilde{M}\left(\rho \right)-\sqrt{{\left(b_{1}-b_{2}\tilde{M}\left(\rho \right)\right)}^{2}-4b_{3}{\tilde{M}}^{3}\left(\rho \right)}\right) & \mathrm{if}\tilde{M}\left(\rho \right)\varepsilon \left[0.448994,0.671256\right)\\ \frac{1}{2c_{3}}\left(c_{2}-\sqrt{c_{2}^{2}+4c_{3}\ln\left(\frac{3-4\tilde{M}\left(\rho \right)}{4c_{1}}\right)}\right) & \mathrm{if}\tilde{M}\left(\rho \right)\varepsilon \left[0.671256,\frac{3}{4}\right) \end{array}\right.$$

with $a_{1}=-0.535707356$, $a_{2}=1.168939911$, $a_{3}=-0.1516189217$, $b_{1}=0.9694429$, $b_{2}=0.8727534$, $b_{3}=0.07350824$, $c_{1}=0.3655157$, $c_{2}=0.6723532$ and $c_{3}=0.033834$. Based on (21) we may apply the following approximation for $\tilde{M}\left(\rho \right)$:

(23) $${\tilde{M}}_{ISI_{j}}\left(\rho \right)≅\frac{{\left(\frac{1}{L}\sum_{i=1}^{i=L}\left|p_{r_{i,ISI_{j}}}\right|\right)}^{2}}{\frac{1}{L}\sum_{i=1}^{i=L}p_{r_{i,ISI_{j}}}^{2}}$$

leading to the notation of ${\tilde{\rho }}_{ISI_{j}}$ for $\tilde{\rho }$. Please note that *L* stands for the total number of Monte Carlo trials. Since *L* may not be very large, the expression for ${\tilde{M}}_{ISI_{j}}\left(\rho \right)$ may be a little too “jumpy”, thereby leading to an incorrect value for $\tilde{\rho }$ in (22). Therefore, we apply some averaging operation on the obtained $\tilde{\rho }$ from (22):

(24) $${\hat{\rho }}_{ISI_{\left[j,j+t-1\right]}}=\frac{1}{t}\sum_{f=0}^{f=t-1}{\tilde{\rho }}_{ISI_{j+f}}$$

where [j,j+t−1] in (24) means that we use *t* samples at each Monte Carlo trial for the averaging operation on $\tilde{\rho }$ (obtained from (22)) which we denoted earlier as ${\tilde{\rho }}_{ISI_{j}}$. Since at each iteration number, the residual ISI is different (is decreasing), the step-size parameter μ in the equalizer’s update mechanism (4) is set in such a way that this difference is relatively small, considering. In other words, the step-size parameter μ is set to be relatively very small. Now, based on (19), (22), (23) and (24), the connection between the approximated shape parameter (an approximation for ρ) and the residual ISI is obtained for a specific channel. Figure 3 shows the approximated shape parameter as a function of the residual ISI in dB units for the 16QAM constellation input sent via three different channels (CH1, CH2 and CH3) for the noiseless case. In addition, the approximated average curve for the three channels as a function of the residual ISI in dB units is also derived and denoted as “Avg”. The three channels (CH1, CH2 and CH3) are defined as follows:

 **CH1**  (initial ISI = 0.88): The channel parameters are determined according to [23]:
  h[n]=[0.4851,−0.72765,−0.4851].
 **CH2**  (initial ISI = 1.402): The channel parameters are determined according to [24]:
  h[n]=[0.2258,0.5161,0.6452,0.5161].
 **CH3**  (initial ISI = 1.715): The channel parameters are based on the carrier serving ares (CSA), loop 1 given in [25], which were down decimated by 32 and normalized so that $h^{T}\left[n\right]h\left[n\right]=1$:
  h[n]=[0.6069,−0.2023,−0.6069,−0.2529,−0.1517,0.0506,0.1011,0.1517,0.2023,0.1517,0.1517,0.1011,0.0506]. The step-size parameter μ was set for channel CH1, CH2 and CH3 to 0.0000027, 0.00001 and 0.0000025 respectively. The equalizer’s tap length *N* was set for channel CH1, CH2 and CH3 to 15, 21 and 57 respectively. Based on the approximated average curve (“Avg”) for the three channels as a function of the residual ISI in dB units, the coefficients of a polynomial P(ISI) of degree four that fit the approximated shape parameter best in a least-squares sense were obtained via the polyfit function from the Matlab software. Thus, we obtained the approximated shape parameter as a polynomial function of the residual ISI in dB units which is given in (15).

Next, we turn to show the various steps that led us to the rest of the equations from the above theorem. As already was mentioned earlier, the convolutional noise pdf is unknown and its shape is changing during the deconvolutional process. Thus, we apply in the following the GGD [16] for approximating the real part of the convolutional noise pdf:

(25) $${\tilde{f}}_{p_{r}}\left(p_{r}\right)=\frac{1}{2\Gamma \left(1+\frac{1}{\rho }\right)B\left(\rho ,\sigma \right)}\exp\left(-|\frac{p_{r}}{B\left(\rho ,\sigma \right)}{|}^{\rho }\right)$$

with

(26) $$B\left(\rho ,\sigma \right)={\left(\frac{\sigma_{p_{r}}^{2}\Gamma \left(\frac{1}{\rho }\right)}{\Gamma \left(\frac{3}{\rho }\right)}\right)}^{\frac{1}{2}}$$

where ${\tilde{f}}_{p_{r}}\left(p_{r}\right)$ is the approximated pdf for $f_{p_{r}}\left(p_{r}\right)$ and ρ is defined as the shape parameter. Please notice, when ρ=1, the GGD (25) corresponds to a Laplacian or double exponential distribution; ρ=2 corresponds to a Gaussian distribution, whereas in the limiting cases ρ→+∞ the pdf in (25) converges to a uniform distribution in $\left(-\sqrt{3}\sigma ,\sqrt{3}\sigma \right)$ [16]. The pdf of the real part of the input sequence is approximated with the maximum entropy density technique [12,13,14,15]:

(27) $${\tilde{f}}_{x_{r}}\left(x_{r}\right)=A\exp\left(\lambda_{2}x_{r}^{2}+\lambda_{4}x_{r}^{4}\right)$$

where ${\tilde{f}}_{x_{r}}\left(x_{r}\right)$ is the approximated pdf for $f_{x_{r}}\left(x_{r}\right)$. $\lambda_{2}$ and $\lambda_{4}$ are the Lagrange multipliers corresponding to the real part of the input sequence pdf and *A* is a constant. According to Bayes rules, the real part of the equalized output pdf is defined as:

(28) $$f_{z_{r}}\left(z_{r}\right)=\int_{-\infty }^{\infty }f\left(z_{r}|x_{r}\right)f_{x_{r}}\left(x_{r}\right)dx_{r}$$

where based on (25)

(29) $$f_{z_{r}|x_{r}}\left(z_{r}|x_{r}\right)≅\frac{1}{2\Gamma \left(1+\frac{1}{\rho }\right)B\left(\rho ,\sigma \right)}\exp\left(-|\frac{z_{r}-x_{r}}{B\left(\rho ,\sigma \right)}{|}^{\rho }\right)$$

with $B\left(\rho ,\sigma \right)$ given in (26). Now, substituting (29) and (27) into (28) and carrying out the integral, an approximated expression for $f_{z_{r}}\left(z_{r}\right)$ is obtained. However, carrying out the integral in (28) is not an easy task, especially when the shape parameter ρ is not known and may even be a fraction. In order to overcome the problem, we use the Edgeworth expansion [17,18,19] up to order six for approximating the approximated pdf for the real part of the convolutional noise pdf (25):

(30) $$\begin{array}{c} {\hat{f}}_{p_{r}}\left(p_{r}\right)=\frac{\exp\left(-\frac{p_{r}^{2}}{2\sigma_{p_{r}}^{2}}\right)}{\sqrt{2\pi }\sigma_{p_{r}}}\left[1+\left(\frac{E\left[p_{r}^{4}\right]-3{\left(\sigma_{p_{r}}^{2}\right)}^{2}}{4!{\left(\sigma_{p_{r}}^{2}\right)}^{2}}\right)\left(\frac{p_{r}^{4}}{{\left(\sigma_{p_{r}}^{2}\right)}^{2}}-\frac{6p_{r}^{2}}{\sigma_{p_{r}}^{2}}+3\right)+\right.\\ \left.\left(\frac{E\left[p_{r}^{6}\right]-15\sigma_{p_{r}}^{2}E\left[p_{r}^{4}\right]+30{\left(\sigma_{p_{r}}^{2}\right)}^{3}}{6!{\left(\sigma_{p_{r}}^{2}\right)}^{3}}\right)\left(\frac{p_{r}^{6}}{{\left(\sigma_{p_{r}}^{2}\right)}^{3}}-\frac{15p_{r}^{4}}{{\left(\sigma_{p_{r}}^{2}\right)}^{2}}+\frac{45p_{r}^{2}}{\sigma_{p_{r}}^{2}}-15\right)\right] \end{array}$$

where ${\hat{f}}_{p_{r}}\left(p_{r}\right)$ is the approximation for ${\tilde{f}}_{p_{r}}\left(p_{r}\right)$. According to [16] we have:

(31) $$E\left[p_{r}^{6}\right]={\left[\frac{\sigma_{p_{r}}^{2}\Gamma \left(\frac{1}{\rho }\right)}{\Gamma \left(\frac{3}{\rho }\right)}\right]}^{3}\frac{\Gamma \left(\frac{7}{\rho }\right)}{\Gamma \left(\frac{1}{\rho }\right)};\;\;\;E\left[p_{r}^{4}\right]={\left[\frac{\sigma_{p_{r}}^{2}\Gamma \left(\frac{1}{\rho }\right)}{\Gamma \left(\frac{3}{\rho }\right)}\right]}^{2}\frac{\Gamma \left(\frac{5}{\rho }\right)}{\Gamma \left(\frac{1}{\rho }\right)}$$

Now, based on (30) we may have for $f_{z_{r}|x_{r}}\left(z_{r}|x_{r}\right)$ the following expression:

(32) $$f_{z_{r}|x_{r}}\left(z_{r}|x_{r}\right)≅\exp\left(\frac{{\left(z_{r}-x_{r}\right)}^{2}}{2\sigma_{p_{r}}^{2}}\right)\left[\tilde{a}+\tilde{b}{\left(z_{r}-x_{r}\right)}^{2}+\tilde{c}{\left(z_{r}-x_{r}\right)}^{4}+\tilde{d}{\left(z_{r}-x_{r}\right)}^{6}\right]$$

with

(33) $$\begin{array}{c} \tilde{a}=\left(1+3\left(\frac{E\left[p_{r}^{4}\right]-3{\left(\sigma_{p_{r}}^{2}\right)}^{2}}{4!{\left(\sigma_{p_{r}}^{2}\right)}^{2}}\right)-15\left(\frac{E\left[p_{r}^{6}\right]-15\sigma_{p_{r}}^{2}E\left[p_{r}^{4}\right]+30{\left(\sigma_{p_{r}}^{2}\right)}^{3}}{6!{\left(\sigma_{p_{r}}^{2}\right)}^{3}}\right)\right)\left(\frac{1}{\sqrt{2\pi }\sigma_{p_{r}}}\right)\\ \tilde{b}=\left(45\left(\frac{E\left[p_{r}^{6}\right]-15\sigma_{p_{r}}^{2}E\left[p_{r}^{4}\right]+30{\left(\sigma_{p_{r}}^{2}\right)}^{3}}{6!{\left(\sigma_{p_{r}}^{2}\right)}^{3}}\right)-6\left(\frac{E\left[p_{r}^{4}\right]-3{\left(\sigma_{p_{r}}^{2}\right)}^{2}}{4!{\left(\sigma_{p_{r}}^{2}\right)}^{2}}\right)\right)\left(\frac{1}{\sigma_{p_{r}}^{2}}\right)\left(\frac{1}{\sqrt{2\pi }\sigma_{p_{r}}}\right)\\ \tilde{c}=\left(\left(\frac{E\left[p_{r}^{4}\right]-3{\left(\sigma_{p_{r}}^{2}\right)}^{2}}{4!{\left(\sigma_{p_{r}}^{2}\right)}^{2}}\right)-15\left(\frac{E\left[p_{r}^{6}\right]-15\sigma_{p_{r}}^{2}E\left[p_{r}^{4}\right]+30{\left(\sigma_{p_{r}}^{2}\right)}^{3}}{6!{\left(\sigma_{p_{r}}^{2}\right)}^{3}}\right)\right)\left(\frac{1}{{\left(\sigma_{p_{r}}^{2}\right)}^{2}}\right)\left(\frac{1}{\sqrt{2\pi }\sigma_{p_{r}}}\right)\\ \tilde{d}=\left(\frac{E\left[p_{r}^{6}\right]-15\sigma_{p_{r}}^{2}E\left[p_{r}^{4}\right]+30{\left(\sigma_{p_{r}}^{2}\right)}^{3}}{6!{\left(\sigma_{p_{r}}^{2}\right)}^{3}}\right)\left(\frac{1}{{\left(\sigma_{p_{r}}^{2}\right)}^{3}}\right)\left(\frac{1}{\sqrt{2\pi }\sigma_{p_{r}}}\right) \end{array}$$

Next, we substitute (32) and (27) into (28) and obtain:

(34) $$f_{z_{r}}\left(z_{r}\right)≅\int_{-\infty }^{+\infty }g\left(x_{r}\right)\exp\left(-\frac{\Psi \left(x_{r}\right)}{\beta }\right)dx_{r}$$

where

(35) $$\begin{array}{c} g\left(x_{r}\right)=A\exp\left(\lambda_{2}x_{r}^{2}+\lambda_{4}x_{r}^{4}\right)\left[\tilde{a}+\tilde{b}{\left(z_{r}-x_{r}\right)}^{2}+\tilde{c}{\left(z_{r}-x_{r}\right)}^{4}+\tilde{d}{\left(z_{r}-x_{r}\right)}^{6}\right]\\ \Psi \left(x_{r}\right)={\left(z_{r}-x_{r}\right)}^{2};\;\;\;\;\beta =2\sigma_{p_{r}}^{2} \end{array}$$

The integral in (34) can be solved with the Laplace’s integral method [20] following [12]:

(36) $$\begin{array}{c} \int_{-\infty }^{+\infty }g\left(x_{r}\right)\exp\left(-\frac{\Psi \left(x_{r}\right)}{\beta }\right)dx_{r}≅\\ \exp\left(-\frac{\Psi \left(x_{0}\right)}{\beta }\right)\sqrt{\frac{2\pi \beta }{\Psi^{{}^{\prime \prime }}\left(x_{0}\right)}}\left(g\left(x_{0}\right)+\frac{g^{{}^{\prime \prime }}\left(x_{0}\right)}{2}\frac{\beta }{\Psi^{{}^{\prime \prime }}\left(x_{0}\right)}+\frac{g^{{}^{⁗}}\left(x_{0}\right)}{8}{\left(\frac{\beta }{\Psi^{{}^{\prime \prime }}\left(x_{0}\right)}\right)}^{2}\right) \end{array}$$

where ${\left(\right)}^{\prime \prime }$ and ${\left(\right)}^{⁗}$ denote the second and fourth derivative of (), respectively. The function $\Psi^{{}^{\prime \prime }}\left(x_{0}\right)$ and $x_{0}$ are obtained via:

(37) $$\begin{array}{c} \Psi^{{}^{\prime }}\left(x_{r}\right)=-2\left(z_{r}-x_{r}\right);\;\;\;\Psi^{{}^{\prime \prime }}\left(x_{r}\right)=2\Rightarrow \Psi^{{}^{\prime \prime }}\left(x_{0}\right)=2;\\ \Psi^{{}^{\prime }}\left(x_{0}\right)=-2\left(z_{r}-x_{0}\right)=0\Rightarrow x_{0}=z_{r} \end{array}$$

By using (37) and (36), the integral in (34) can be written as:

(38) $$f_{z_{r}}\left(z_{r}\right)≅\sqrt{\pi \beta }\left(g\left(x_{0}\right)+\frac{g^{{}^{\prime \prime }}\left(x_{0}\right)}{2}\frac{\beta }{2}+\frac{g^{{}^{⁗}}\left(x_{0}\right)}{8}{\left(\frac{\beta }{2}\right)}^{2}\right)$$

with

(39) $$\begin{array}{c} g\left(x_{0}\right)=A\exp\left(\lambda_{2}z_{r}^{2}+\lambda_{4}z_{r}^{4}\right)\tilde{a}\\ g^{{}^{\prime \prime }}\left(x_{0}\right)=A\exp\left(\lambda_{2}z_{r}^{2}+\lambda_{4}z_{r}^{4}\right)\left(\tilde{a}{\left(2\lambda_{2}z_{r}+4\lambda_{4}z_{r}^{3}\right)}^{2}+\tilde{a}\left(2\lambda_{2}+12\lambda_{4}z_{r}^{2}\right)+2\tilde{b}\right)\\ g^{{}^{⁗}}\left(x_{0}\right)=A\exp\left(\lambda_{2}z_{r}^{2}+\lambda_{4}z_{r}^{4}\right)\left(\tilde{a}{\left(2\lambda_{2}z_{r}+4\lambda_{4}z_{r}^{3}\right)}^{4}+6\tilde{a}\left(2\lambda_{2}+12\lambda_{4}z_{r}^{2}\right){\left(2\lambda_{2}z_{r}+4\lambda_{4}z_{r}^{3}\right)}^{2}+\right.\\ \left.12\tilde{b}{\left(2\lambda_{2}z_{r}+4\lambda_{4}z_{r}^{3}\right)}^{2}+3\tilde{a}{\left(2\lambda_{2}+12\lambda_{4}z_{r}^{2}\right)}^{2}+96\tilde{a}\lambda_{4}z_{r}\left(2\lambda_{2}z_{r}+4\lambda_{4}z_{r}^{3}\right)+\right.\\ \left.12\tilde{b}\left(2\lambda_{2}+12\lambda_{4}z_{r}^{2}\right)+24\tilde{a}\lambda_{4}+24\tilde{c}\right) \end{array}$$

Another way to obtain the approximated pdf of the real part of the equalized output sequence is via the maximum entropy density approximation technique [12,13,14,15]:

(40) $$f_{z_{r}}\left(z_{r}\right)≅A\exp\left({\tilde{\lambda }}_{2}z_{r}^{2}+{\tilde{\lambda }}_{4}z_{r}^{4}\right)$$

where the Lagrange multipliers ${\tilde{\lambda }}_{2}$ and ${\tilde{\lambda }}_{4}$ are calculated according to (17) and (18). On average, both the approximated expressions for the pdf of the real part of the equalized output sequence (40) and (38) should give the same results. Thus using (40) and (38), we may write:

(41) $$E\left[A\exp\left({\tilde{\lambda }}_{2}z_{r}^{2}+{\tilde{\lambda }}_{4}z_{r}^{4}\right)-\sqrt{\pi \beta }\left(g\left(x_{0}\right)+\frac{g^{{}^{\prime \prime }}\left(x_{0}\right)}{2}\frac{\beta }{2}+\frac{g^{{}^{⁗}}\left(x_{0}\right)}{8}{\left(\frac{\beta }{2}\right)}^{2}\right)\right]=0$$

Next we may write with the help of [26]:

(42) $$\begin{array}{c} A\exp\left({\tilde{\lambda }}_{2}z_{r}^{2}+{\tilde{\lambda }}_{4}z_{r}^{4}\right)=A\exp\left(\lambda_{2}z_{r}^{2}+\lambda_{4}z_{r}^{4}\right)\exp\left(\Delta \lambda_{2}z_{r}^{2}+\Delta \lambda_{4}z_{r}^{4}\right)≅\\ A\exp\left(\lambda_{2}z_{r}^{2}+\lambda_{4}z_{r}^{4}\right)\left(1+\left(\Delta \lambda_{2}z_{r}^{2}+\Delta \lambda_{4}z_{r}^{4}\right)+\frac{1}{2}{\left(\Delta \lambda_{2}z_{r}^{2}+\Delta \lambda_{4}z_{r}^{4}\right)}^{2}+\frac{1}{6}{\left(\Delta \lambda_{2}z_{r}^{2}+\Delta \lambda_{4}z_{r}^{4}\right)}^{3}\right) \end{array}$$

where $\Delta \lambda_{2}$ and $\Delta \lambda_{4}$ are defined in (16). In addition, with the help of (31), (33), (35) and (39), we have:

(43) $$\sqrt{\pi \beta }\left(g\left(x_{0}\right)+\frac{g^{{}^{\prime \prime }}\left(x_{0}\right)}{2}\frac{\beta }{2}+\frac{g^{{}^{⁗}}\left(x_{0}\right)}{8}{\left(\frac{\beta }{2}\right)}^{2}\right)=A\exp\left(\lambda_{2}z_{r}^{2}+\lambda_{4}z_{r}^{4}\right)\left(a+\frac{S_{1}}{2}\sigma_{p_{r}}^{2}+\frac{S_{2}}{8}{\left(\sigma_{p_{r}}^{2}\right)}^{2}\right)$$

where

(44) $$\begin{array}{c} S_{1}=16a\lambda_{4}^{2}z_{r}^{6}+16a\lambda_{2}\lambda_{4}z_{r}^{4}+\left(4a\lambda_{2}^{2}+12a\lambda_{4}\right)z_{r}^{2}+2b+2a\lambda_{2}\\ S_{2}=256a\lambda_{4}^{4}z_{r}^{12}+512a\lambda_{2}\lambda_{4}^{3}z_{r}^{10}+\left(384a\lambda_{2}^{2}\lambda_{4}^{2}+1152a\lambda_{4}^{3}\right)z_{r}^{8}+\\ \left(128a\lambda_{2}^{3}\lambda_{4}+1344a\lambda_{2}\lambda_{4}^{2}+192b\lambda_{4}^{2}\right)z_{r}^{6}+\left(816a\lambda_{4}^{2}+480a\lambda_{2}^{2}\lambda_{4}+16a\lambda_{2}^{4}+192b\lambda_{2}\lambda_{4}\right)z_{r}^{4}+\\ \left(144b\lambda_{4}+48a\lambda_{2}^{3}+336a\lambda_{4}\lambda_{2}+48b\lambda_{2}^{2}\right)z_{r}^{2}+12a\lambda_{2}^{2}+24b\lambda_{2}+24a\lambda_{4}+24c \end{array}$$

and *a*, *b* and *c* are defined in (12). Now we put (43) and (42) into (41) and obtain:

(45) $$\begin{array}{c} E\left[A\exp\left(\lambda_{2}z_{r}^{2}+\lambda_{4}z_{r}^{4}\right)\left(1+\left(\Delta \lambda_{2}z_{r}^{2}+\Delta \lambda_{4}z_{r}^{4}\right)+\frac{1}{2}{\left(\Delta \lambda_{2}z_{r}^{2}+\Delta \lambda_{4}z_{r}^{4}\right)}^{2}+\frac{1}{6}{\left(\Delta \lambda_{2}z_{r}^{2}+\Delta \lambda_{4}z_{r}^{4}\right)}^{3}-\right.\right.\\ \left.\left.\left(a+\frac{S_{1}}{2}\sigma_{p_{r}}^{2}+\frac{S_{2}}{8}{\left(\sigma_{p_{r}}^{2}\right)}^{2}\right)\right)\right]=0 \end{array}$$

which leads to:

(46) $$\begin{array}{c} E\left[1+\left(\Delta \lambda_{2}z_{r}^{2}+\Delta \lambda_{4}z_{r}^{4}\right)+\frac{1}{2}{\left(\Delta \lambda_{2}z_{r}^{2}+\Delta \lambda_{4}z_{r}^{4}\right)}^{2}+\frac{1}{6}{\left(\Delta \lambda_{2}z_{r}^{2}+\Delta \lambda_{4}z_{r}^{4}\right)}^{3}\right]≅\\ E\left[a+\frac{S_{1}}{2}\sigma_{p_{r}}^{2}+\frac{S_{2}}{8}{\left(\sigma_{p_{r}}^{2}\right)}^{2}\right] \end{array}$$

By using (44) and carrying out the expectation operator in (46) we obtain (11). □

## 3. Simulation

In this section, we test our proposed approximated expression for the ISI (11) valid for the 16QAM input sequence. For that case, the equalizer is initialized by setting the center tap equal to one and all others to zero. In addition, the step-size parameter μ is set to zero which means that the equalizer’s coefficients are not updated. Since the expression for the initial ISI (11) is a function of the moments of the real part of the equalized output sequence, we first wish to see how different sizes of samples from the real part of the equalized output sequence influence on the approximated expression for the ISI (11). For simplicity, we denote “K” as the amount of samples participating in the calculations of the various moments of the real part of the equalized output sequence. Although the approximated ISI (11) was derived for the noiseless case, it will be tested in the following also for SNR values of 30 and 20 dB. In the following we denote $ISI_{cal}$ and $ISI_{sim}$ as the approximated ISI calculated via (11) and the simulated ISI respectively. Table 1, Table 2, Table 3, Table 4, Table 5, Table 6, Table 7, Table 8, Table 9, Table 10, Table 11, Table 12, Table 13, Table 14 and Table 15 show the performance of $ISI_{cal}$ compared with $ISI_{sim}$ for nine different channels, with different sizes of *K* and with two different values for the SNR (SNR=30db, SNR=20db). The nine different channels are defined as:

 **CH1**  (Initial ISI = 0.88): The channel parameters are determined according to [23]:
  h[n]=[0.4851,−0.72765,−0.4851].
 **CH2** (Initial ISI = 1.402): The channel parameters are determined according to [24]:
  h[n]=[0.2258,0.5161,0.6452,0.5161].
 **CH3**  (Initial ISI = 1.715): The channel parameters are based on the carrier serving area (CSA) loop 1 given in [25] which were down decimated by 32 and normalized so that $h^{T}\left[n\right]h\left[n\right]=1$:
  h[n]=[0.6069,−0.2023,−0.6069,−0.2529,−0.1517,0.0506,0.1011,0.1517,0.2023,0.1517,0.1517,0.1011,0.0506].
 **CH4**  (Initial ISI = 0.389): The channel parameters are determined according to:
  h[n]=[0.3842,0.8704,0.3842].
 **CH5**  (Initial ISI = 0.73): The channel parameters are determined according to:
  h[n]=[1,0.8,0.3].
 **CH6**  (Initial ISI = 1): The channel parameters are determined according to:
  h[n]=[1,0.8,0.6].
 **CH7**  (Initial ISI = 0.41): The channel parameters are determined according to:
  h[n]=[0.5,1,0.4].
 **CH8**  (Initial ISI = 1.13): The channel parameters are determined according to:
  h[n]=[1,0.8,0.7].
 **CH9**  (Initial ISI = 1.395): The channel parameters are determined according to:
  h[n]=[0.9,0.8,0.7].

According to Table 1, Table 2, Table 3, Table 4 and Table 5, there is no need to apply a large number of samples of the real part of the equalized output sequence for calculating the various moments appearing in (11). According to Table 1, Table 2, Table 3, Table 4 and Table 5, the obtained value for $ISI_{cal}$ is very close to the obtained value for $ISI_{sim}$ for CH2, while the difference between the values for $ISI_{cal}$ and $ISI_{sim}$ for CH1 and CH3 is higher. But still, according to Table 1, Table 2, Table 3, Table 4 and Table 5, we are able to say which are the easiest and worst channels from the ISI point of view. Thus, we can choose to select the easiest channel which is in this case CH1 with an initial ISI of 0.88 and not the worst channel (CH3) with initial ISI of 1.715. In addition, we see that although $ISI_{cal}$ was derived for the noiseless case, it works also for SNR values down to 20 db.

The expression for $ISI_{cal}$ depends on (15) which was obtained with the help of channel CH1, CH2 and CH3, as explained earlier in the previous section. Thus, next we wish to test the performance of $ISI_{cal}$ with other channels than only with CH1, CH2 and CH3. Table 6, Table 7, Table 8, Table 9, Table 10, Table 11, Table 12, Table 13, Table 14 and Table 15 show the performance of $ISI_{cal}$ compared with $ISI_{sim}$ for various values for *Q*, *K*, types of channels and values for SNR. According to Table 6, Table 7, Table 8, Table 9, Table 10, Table 11, Table 12, Table 13, Table 14 and Table 15, a very high correlation is obtained between the calculated ISI ($ISI_{cal}$) and the simulated one ($ISI_{sim}$). This means that if we apply the right value for *Q* for the chosen channels, then excellent performance from the ISI point of view can be obtained from $ISI_{cal}$, even down to SNR values of 20db. Please note, according to Table 8, the same *Q* is applied for a very easy channel (CH4) and for a much harder channel (CH5) having approximately twice the intial ISI of CH4.

The Laplace’s integral method [12,20] used in (36) is a general technique for obtaining the asymptotic behavior as β→0 of integrals in which the large parameter 1/β appears in the exponent. It turns out in practice that analysis that is based on low noise, making the Laplace integral and singular perturbation method feasible, can be extended to the region where the noise is not low. Note, for example, the papers [27,28] wherein the Laplace integral and the singular perturbation method were applied under low noise assumption and the results could be very well extended to the medium and high noise range (as a matter of fact, these methods were rather successful in calculating even the threshold region) [12]. That is also the case in this paper where good results are obtained for the very high ISI condition.

## 4. Conclusions

In this paper, we proposed a systematic approach for achieving the approximated ISI from each sub-channel modeled as a FIR channel with real-valued coefficients for a 16QAM source signal transmission. The approximated ISI is based on the maximum entropy density approximation technique, on the Edgeworth expansion up to order six, on the Laplace integral method and on the GGD. Although the approximated ISI was derived for the noiseless case, it was successfully tested for SNR values down to 20 dB. As a by-product, we obtained a new presentation for the real part of the convolutional noise pdf based on the GGD where the shape parameter is a function of the residual ISI. Thus, the real part of the convolutional noise pdf has the ability of changing its shape during the iterative deconvolution process. Therefore, it might be useful in the derivation of a new approximated closed-form expression for the conditional expectation (the expectation of the input signal given the equalized output sequence), associated with the blind adaptive deconvolution problem, that will be carried out in a future work. It should be pointed out that the approximated expression for the ISI, proposed in this paper, can be applied also to other input sequences where the real and imaginary parts of the input signal are independent, as long as the expression for the shape parameter as a function of the ISI is given for this new input sequence, and the approximated input pdf can be modeled with the maximum entropy density approximation technique with Lagrange multipliers up to order four. Thus, this paper can be extended with a new derivation for the shape parameter as a function of the ISI for the general input case where the real and imaginary parts of the input signal are independent and where the approximated input pdf can be modeled with the maximum entropy density approximation technique with Lagrange multipliers up to order four.

## Figures and Tables

**Figure 2:**
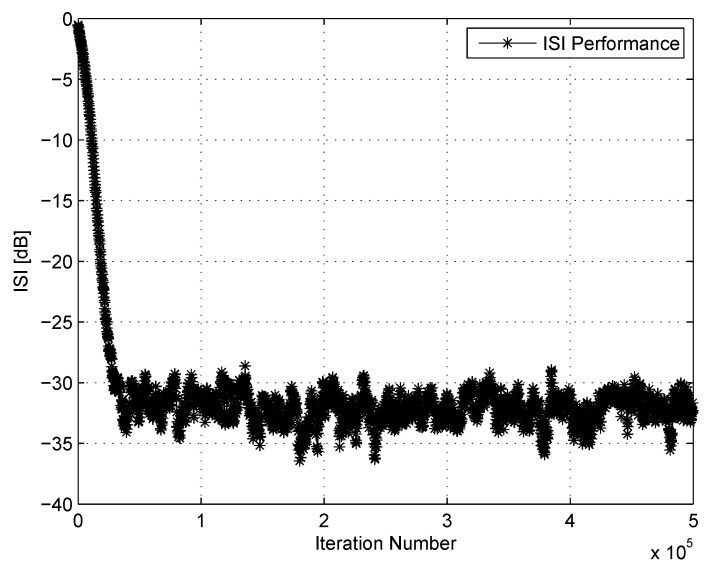
ISI performance for CH2.

**Table 1:** Performance of the approximated inter-symbol-interference (ISI) (11) obtained after 100 Monte Carlo trials.

	Q = 0.2; K = 2000; Noiseless Case	
	$ISI_{cal}$	$ISI_{sim}$
CH1	1.1271	0.88
CH2	1.3604	1.402
CH3	1.5219	1.715

**Table 2:** Performance of the approximated ISI (11) obtained after 100 Monte Carlo trials.

	Q = 0.2; K = 2000; SNR = 30 dB	
	$ISI_{cal}$	$ISI_{sim}$
CH1	1.1241	0.88
CH2	1.3916	1.402
CH3	1.5505	1.715

**Table 3:** Performance of the approximated ISI (11) obtained after 100 Monte Carlo trials.

	Q = 0.2; K = 2000; SNR = 20 dB	
	$ISI_{cal}$	$ISI_{sim}$
CH1	1.1355	0.88
CH2	1.3798	1.402
CH3	1.5166	1.715

**Table 4:** Performance of the approximated ISI (11) obtained after 100 Monte Carlo trials.

	Q = 0.2; K = 4000; SNR = 20 dB	
	$ISI_{cal}$	$ISI_{sim}$
CH1	1.2199	0.88
CH2	1.4019	1.402
CH3	1.5559	1.715

**Table 5:** Performance of the approximated ISI (11) obtained after 100 Monte Carlo trials.

	Q = 0.2; K = 10,000; SNR = 20 dB	
	$ISI_{cal}$	$ISI_{sim}$
CH1	1.2217	0.88
CH2	1.4153	1.402
CH3	1.5684	1.715

**Table 6:** Performance of the approximated ISI (11) obtained after 100 Monte Carlo trials.

	Q = 0.26; K = 2000; SNR = 30 dB	
	$ISI_{cal}$	$ISI_{sim}$
CH1	0.8728	0.88
CH9	1.3986	1.395

**Table 7:** Performance of the approximated ISI (11) obtained after 100 Monte Carlo trials.

	Q = 0.26; K = 2000; SNR = 20 dB	
	$ISI_{cal}$	$ISI_{sim}$
CH1	0.9358	0.88
CH9	1.4036	1.395

**Table 8:** Performance of the approximated ISI (11) obtained after 100 Monte Carlo trials.

	Q = 0.46; K = 2000; SNR = 30 dB	
	$ISI_{cal}$	$ISI_{sim}$
CH4	0.3302	0.389
CH5	0.7478	0.73

**Table 9:** Performance of the approximated ISI (11) obtained after 100 Monte Carlo trials.

	Q = 0.46; K = 2000; SNR = 20 dB	
	$ISI_{cal}$	$ISI_{sim}$
CH4	0.3832	0.389
CH5	0.7471	0.73

**Table 10:** Performance of the approximated ISI (11) obtained after 100 Monte Carlo trials.

	Q = 0.34; K = 2000; SNR = 30 dB	
	$ISI_{cal}$	$ISI_{sim}$
CH6	1.0763	1
CH8	1.0938	1.13

**Table 11:** Performance of the approximated ISI (11) obtained after 100 Monte Carlo trials.

	Q = 0.34; K = 2000; SNR = 20 dB	
	$ISI_{cal}$	$ISI_{sim}$
CH6	1.0792	1
CH8	1.0966	1.13

**Table 12:** Performance of the approximated ISI (11) obtained after 100 Monte Carlo trials.

	Q = 0.35; K = 2000; SNR = 30 dB	
	$ISI_{cal}$	$ISI_{sim}$
CH6	1.0461	1
CH8	1.0631	1.13

**Table 13:** Performance of the approximated ISI (11) obtained after 100 Monte Carlo trials.

	Q = 0.35; K = 2000; SNR = 20 dB	
	$ISI_{cal}$	$ISI_{sim}$
CH6	1.0489	1
CH8	1.0658	1.13

**Table 14:** Performance of the approximated ISI (11) obtained after 100 Monte Carlo trials.

	Q = 0.76; K = 2000; SNR = 30 dB	
	$ISI_{cal}$	$ISI_{sim}$
CH7	0.4085	0.41

**Table 15:** Performance of the approximated ISI (11) obtained after 100 Monte Carlo trials.

	Q = 0.76; K = 2000; SNR = 20 dB	
	$ISI_{cal}$	$ISI_{sim}$
CH7	0.4162	0.41

## Abbreviations

The following abbreviations are used in this manuscript:

ISI	Inter-Symbol-Interference
GGD	Generalized Gaussian Distribution
FIR	Finite Impulse Response
SIMO	Single Input Multiple Output
16QAM	16 Quadrature Amplitude Modulation
pdf	Probability Density Function
CSA	Carrier Serving Area
